# Skin inflammatory reactions to capsaicin in rheumatoid arthritis patients compared to healthy controls

**Published:** 2019

**Authors:** Masoumeh Salari, Roshanak Salari, Houshang Rafatpanah, Yalda Ravanshad, Danial Zirachi, Maryam Sahebari

**Affiliations:** 1 *Rheumatic Diseases Research Center, Mashhad University of Medical Sciences, Mashhad, Iran*; 2 *Department of Traditional Persian Pharmacy, School of Persian and Complementary Medicine, Mashhad University of Medical Sciences, Mashhad, Iran*; 3 *Allergy Research Center, Mashhad University of Medical Sciences, Mashhad, Iran*; 4 *Clinical Research Unit, Mashhad University of Medical Sciences, Mashhad, Iran*

**Keywords:** Rheumatoid arthritis, Capsaicin, Inflammation

## Abstract

**Objective:**

Previous studies have examined the role of sensory nerves and neural mediators in symmetrical joint inflammation and autoimmunity in rheumatoid arthritis (RA). In this study, we sought to examine the association between capsaicin skin test and disease activity in RA patients.

**Materials and Methods:**

Eighty RA patients (case group) and 20 healthy volunteers were enrolled in this experiment. The subjects in case group were calcified to newly diagnosed patients and those previously diagnosed with RA. A topical solution of capsaicin (0.075%) was applied on the volar forearm during the skin test, and evaluations were done after 5, 10, and 20 min. The parameters recorded in capsaicin skin test were time to tingling, area of induration (cm^2^), and area of redness (cm^2^).

**Results:**

A faster capsaicin skin reaction was observed in healthy controls compared to the case group (p=0.02). Newly diagnosed and previously diagnosed RA patients were not significantly different in terms of reaction latency (p=0.06). The redness area after 15 (p=0.04) and 20 (p=0.001) min was significantly larger in previously diagnosed RA patients than in newly diagnosed ones. The ​​area of redness and time to tingling did not show any difference between active and inactive RA patients, but after 15 and 20 min, the area of redness was significantly greater in inactive RA patients compared to active RA patients (p=0.01 and p=0.03, respectively).

**Conclusion:**

This study suggested that capsaicin skin test is not an efficient tool for the examination of synovial inflammation and disease activity in RA.

## Introduction

Rheumatoid arthritis (RA) is a chronic autoimmune polyarthritis with joint deformity. Despite several approaches suggested for RA treatment, treatment of pain and non-responsiveness are still the most important challenges in this disease (McInnes and Schett, 2017 ▶). 

Different theories have suggested the role of the peripheral (PNS) and central nervous system (CNS) and peri-articular tissues in joints pain (Arendt-Nielsen et al., 2015 ▶). Recent experimental studies have shed light on the role of peripheral nerves in arthritic pain; however, their role have not been investigated in human studies (Borbély et al., 2016 ▶). 

Several studies have shown the effect of noxious and non-noxious stimuli in increasing peripheral nervous system activity in RA. The transient receptor potential vanilloid receptor (TRPV1) is a part of the inflammation pathway in various parts of the body with an inflammatory response and this response is significantly reduced in the absence of TRPV1 ( Hong et al., 2008 ▶; Horváth et al., 2016 ▶; Knotkova et al., 2008 ▶; Romac et al., 2008 ▶). 

TRPV1 has been recognized in human RA synovial fibroblasts by immunohistochemical examinations (Kelly et al., 2015 ▶). As a result, it is hypothesized that TRPV1agonists may activate non-neuronal mechanisms that might modulate nociception in symptomatic RA patients (Engler et al., 2007 ▶).

Capsaicin activates non-neuronal TRPV1, which leads to the release of interleukin (IL-)8, IL-6, and prostaglandin E2 (PGE2) (Seki et al., 2007 ▶). Capsaicin, which can be found in red pepper and derived from oleoresin capsicum and capsicum, is an agonist of TRPV1. The analgesic effects of capsaicin is mediated through localized depletion of substance P, a neuropeptide that transmits pain from the CNS to the PNS (Lin et al., 2004 ▶; Pigatto et al., 2017 ▶; Prescott and Julius, 2003 ▶). In TRPV1-deficient mice , capsaicin-induced nocifensive behavior is disrupted, thus, it seems that capsaicin-induced pain depends on TRPV1 activation (Caterina et al., 2000 ▶). It has been shown that the stimulation caused by capsaicin in the early stages of arthritis produces chemical mediators and substance P, which are involved in induction of pain in the inflamed joints (Engler et al., 2007 ▶). 

 Prescott et al. showed that by blocking neuropeptides such as substance P, neurogenic inflammation is suppressed in RA (Prescott and Julius, 2003 ▶). Walsh et al. reported that perivascular myelin-free nerve fibers and synovial tissue release substance P to control synovial inflammatory processes (Walsh et al., 1992 ▶). Further studies confirmed reduced pain and inflammation in joints by reduction in substance P upon nonsteroidal anti-inflammatory therapy (Sacerdote et al., 1995 ▶). Further, several studies have reported higher plasma and synovial levels of substance P in a variety of inflammatory arthritis patients compared to healthy controls (Menkes et al., 1993 ▶; Wayne et al., 1990 ▶).

The role of capsaicin in reduction of joint tenderness and pain has been shown in experimental models. These models emphasize the role of the CNS in multiple joint involvements (Casanueva et al., 2013 ▶). It has been found that capsaicin reduces the sensitivity of sensory neurons and decreases pain (O'Connor et al., 2004 ▶).

This study was designed to assess skin reaction to capsaicin as an indicator of substance P release, in RA patients and compare it with that of healthy controls. 

## Materials and Methods

This case-control study was performed on 20 healthy controls and 80 RA patients selected based on the standard clinical criteria. The exclusion criteria were hypersensitivity to pepper, taking analgesic and nonsteroidal anti-inflammatory drugs) NSAIDs), peripheral neuropathy, diabetes, asthma, pregnancy, lactation, and known skin diseases like psoriasis or pemphigus.

In general, 80 RA patients (40 newly diagnosed and 40 with previously diagnosed RA) were recruited from patients visiting the Rheumatic Diseases Research Center affiliated to Mashhad University of Medical Sciences, Mashhad, Iran, in 2014. Newly diagnosed patients were RA patients who were recently diagnosed according to the American College of Rheumatology (ACR) criteria, and enrolled into the study before capsaicin treatment. 

Previously diagnosed RA patients were diagnosed for at least one year and were receiving the standard treatment. Disease Activity Score-28 for Rheumatoid Arthritis with ESR (DAS28ESR) was used to measure RA disease activity with DAS28ESR>2.5 defined as active disease.

 The control group included 20 healthy age- and gender-matched volunteers from Ghaem Hospital staff that were selected following a careful physical examination and an interview about medications.

Previously diagnosed RA patients were receiving 5-10 mg prednisolone, 7.5-15 mg/week methotrexate, 1000 mg/d calcium, and 70 mg/week alendronate. 

The patients were first placed in a comfortable position for 15 min. For each participant, a 1×1 cm^2^ blotting paper imbrued with 0.1 ml of capsaicin solution (0.075%; Sigma Company, US) was placed on the volar forearm and covered by a plastic band to prevent evaporation during the skin test.Time to tingling(itching and burning), which was evaluated subjectively, was then recorded. Redness area at the site of skin test was assessed and measured after10, 15, and 20min with transparent paper (oily paper) on the red area, and the area surrounding redness was marked and measured. The redness area was assessed by the AutoCAD software at each step.

For the measurement of indurated area after 20 min, the method of tuberculin skin test was used. The maximum diameter of swelling was recorded. 

**Ethical considerations**

This study was approved by the Ethics Committee of Mashhad University of Medical Sciences, Mashhad, Iran. All the ethical considerations, including the confidentiality of the data as well as the stages and techniques of this study were clearly explained to the patients and informed consent forms were signed by the patients.

**Statistical analysis**

After entering the data into SPSS, version 11.5, descriptive and analytical statistics were used. Kolmogorov-Smirnov test was run for examining the normality of variables, including age, body mass index, disease activity score, visual analogue scale (VAS) score, time to tingling, redness area after 5, 15 and 20 min, area of induration, erythrocyte sedimentation rate (ESR), and duration of disease and these parameters were compared between the two groups. The distributions of time to tingling, redness area after 10 min, induration area, ESR, and course of the disease were not normal. In addition, the linear regression model was used to analyze the data. A p-value less than 0.05 was considered statistically significant.

## Results

Eighty RA patients and 20 healthy volunteers were assessed for skin response to capsaicin. Among the RA patients, 10 (12.5%) were male and 70 (87.5%) were female. In the healthy volunteers group, 5 (25%) were male and 15 (75%) were female; the two groups were not significantly different in this regard (χ^2^=1.96, p=0.16).

**Comparison of RA patients with healthy controls**

The mean ages of the RA and control groups were 43.95±14.79 and 43.80±13.93 years, respectively (t=0.41, p=0.96). The mean body mass index (BMI) was26.63±4.24 and 25.90±5.19 kg/m^2^ in RA and control groups, respectively showing no significant difference between the two groups (t=0.65, p=0.51). Mean disease activity score (DAS) was 4.8±1.7 in RA patients. We also found that time to tingling was significantly shorter in healthy controls. Data obtained from the capsaicin skin test are summarized in Table 1. 

**Table 1:** Capsaicin Skin Test in RA Patients and Controls

	**Therapeutic Group**	**No.**	**Mean**	**SD**	**Statistics test **	**p-value**
**Time to tingling** **(min)**	Control	20	6.65	5	Z=2.25	0.02*
	Patient	79	9.54	8		
	Total	99	8.96	8		
**Redness area after 10 min** **(cm** ^2^ **)**	Control	20	16.70	22.1	Z=1.54	0.12
	Patient	80	11.39	7.8		
	Total	100	12.45	8.65		
**Redness area after 15 min** **(cm** ^2^ **)**	Control	20	25.46	29.65	t=1.64	0.104
	Patient	80	18.17	14.68		
	Total	100	19.63	15.35		
**Redness area after 20 min** **(cm** ^2^ **)**	Control	20	33.10	45.7	t=1.41	0.161
	Patient	80	25.61	19.61		
	Total	100	27.11	22		
**Mean redness area** **(cm** ^2^ **)**	Control	20	24.90	31.73	Z=1.52	0.12
	Patient	80	20.28	14.35		
	Total	100	21.20	15.85		
**Induration** **(cm** ^2^ **)**	Control	20	2.57	1	Z=0.47	0.63
	Patient	80	3.96	2.13		
	Total	100	3.68	1.94		

**Comparison of newly and previously diagnosed RA patients**

Capsaicin test results were compared between newly and previously diagnosed RA patients. Time to tingling was not significantly different between the two groups (Z=-1.88, p=0.06). Redness area in previously diagnosed RA patients at 15 (Z=2.05, p=0.04) and 20(Z=3.612, p=0.001) min post-application was greater than that of the new patients.

**Study of patients based on DAS28ESR**

RA patients were divided into two groups inactive (DAS28ESR≤2.5) and active (DAS28ESR>2.5) patients. There were 7 patients in the DAS28ESR≤2.5 group with the mean age of 41.1±10.6 years and 73 patients in the DAS28ESR>2.5 group with the mean age of 44.2±15.1 years (p=0.4). 

Table 2 presents capsaicin test results in RA patients. Based on the obtained results, ​​redness area and time to tingling did not significantly vary between active and inactive patients. However, after 15 (p=0.014, t test) and 20 (p=0.033, t test) min, redness areas were larger in the inactive group than in the active patients. In addition, there was no statistically significant difference in indurated area between the two groups. 

**Table 2:** Capsaicin Skin Test Based on the Disease Activity Score (DAS28ESR) in RA

	**DAS**	**No.**	**Mean**	**SD**	**Statistics test **	**p-value**
**Time to tingling(min)**	DASESR≤2.5	7	9.85	10.05	Z=0.407	0.68
	DASESR>2.5	72	9.51	7.03		
	Total	79	9.54	7.26		
**Redness area after 10 min(cm** ^2^ **)**	DASESR≤2.5	7	16.85	23.22	Z=0.13	0.89
	DASESR>2.5	73	10.87	11.71		
	Total	80	11.39	12.99		
**Redness area after 15 min(cm** ^2^ **)**	DASESR≤2.5	7	27.38	26.66	t=2.49	0.01*
	DASESR>2.5	73	17.28	16.38		
	Total	80	18.17	17.51		
**Redness area after 20 min(cm** ^2^ **)**	DASESR≤2.5	7	40.14	39.6	t=2.21	0.03*
	DASESR>2.5	73	24.21	18.33		
	Total	80	25.6	21.11		
**Mean redness area(cm** ^2^ **)**	DASESR≤2.5	7	28.1	28.6	Z=0.53	0.59
	DASESR>2.5	73	19.52	22.39		
	Total	80	20.27	22.91		
**Induration(cm** ^2^ **)**	DASESR≤2.5	7	3.33	3.93	Z=0.05	0.98
	DASESR>2.5	73	4.02	6.2		
	Total	80	3.96	6.02		

**Drug influence on capsaicin test**

About 37% of RA patients were receiving prednisolone, 27% were receiving methotrexate (MTX), 26% were receiving hydroxychloroquine, and 11% were receiving sulfasalazine.

A significant positive correlation was found between prednisone (mg/day; p<0.001, r_p_=0.5) and methotrexate (mg/week; p<0.001, r_p_=0.38) doses and time to tingling in RA patients. 

**The results of the regression model**

*A: Skin reaction to capsaicin in terms of redness area after 10, 15, and 20 minutes*

The relationship between the ​​redness area at different time points with new and old case variables was not statistically significant after 10 and 15 min (p=0.44 and0.656, respectively), but this relationship was significant after 20 min (p=0.001), suggesting an increase in the mean area of ​​redness in previously diagnosed RA cases at 20 min. 

To investigate the relationship between the dependent variable (redness area) at the 20^th^ minute and independent variables (i.e., age, gender, BMI, DAS, newly diagnosed RA, history of red pepper consumption, and prednisone and methotrexate doses), multiple linear regression model was used and ENTER method was used for variables choice. In the assessment of the model, (R=0.478, R square = 0.228 and adjusted R square=0.141, B=47.335, p=0.01) was measured. According to R square=0.228, the power of the model to predict the dependent variable based on the independent variables was 22.8%, which is less than 80%. 

Additionally, newly diagnosed disease, as an independent variable significantly correlated with area of redness after 20 min (p=0.005; Table 3). It means that the area of redness after 20 min in newly diagnosed RA patients was smaller than that of old cases.

**Table 3:** Relationship between the dependent variable "redness area after 20 min" and independent variables in linear regression model

**Model**	**Unstandardized Coefficients**		**Standardized Coefficients**	**t**	**p-value**
	**B**	**Std. Error**	**Beta**		
**1 (Constant)**	47.335	19.703		2.402	.019
**DAS28ESR** *	-2.112	1.489	-.173	-1.418	.161
**New Case**	-18.472	6.402	-.440	-2.885	.005
**Age**	-.105	.160	-.074	-.659	.512
**sex**	-.245	6.959	-.004	-.035	.972
**BMI** **	.165	.558	.033	.296	.768
**Red Pepper consumption**	2.156	5.016	.045	.430	.669
**Prednisolone dose(mg/d) **	-.603	.431	-.193	-1.398	.166
**MTX** *** ** dose(mg/w) **	-.462	.826	-.090	-.559	.578

* Disease Activity Score-Erythrocyte Sedimentation Rate

** Body mass index

*** Methotrexate.

Mean area of redness in new cases was 10.74±10.3 cm^2^, and in the previously diagnosed RA cases, it was 12.04±15.3 cm^2^ after 20 min considering confounding variables.

*B: Skin reaction to capsaicin in terms of induration area*

The power of multiple linear regression models in assessing the relationship between the dependent variable “indurated area” and independent variables (i.e., age, gender, BMI, DAS, new case, history of pepper consumption, and prednisone and methotrexate doses) was 9.1%, which is not satisfactory. ANOVA did not reflect any relationship between the dependent variable “indurated area” and independent variables in the linear regression model (p=0.7; Table 4).

The mean indurated area showed no statistically significant difference (p=0.821) in the two groups of previously diagnosed RA patients (3.80±4.18 mm) and new cases (4.11±7.48 mm).

**Table 4:** Relationship between the dependent variable “induration area” and independent variables in the linear regression models

**Model**	**Unstandardized Coefficients**		**Standardized Coefficients**	**t**	**p-value**
	**B**	**Std. Error**	**Beta**		
**1 (Constant)**	1.869	6.100		.306	.760
**DAS28ESR** *	-.843	.461	-.242	-1.827	.072
**New Case**	1.246	1.982	.104	.628	.532
**Age**	-.021	.049	-.051	-.422	.675
**Sex**	1.064	2.155	.059	.494	.623
**BMI** **	.231	.173	.163	1.338	.185
**Red Pepper consumption**	-1.805	1.553	-.133	-1.162	.249
**mg/d Prednisolone dose**	-.136	.134	-.152	-1.019	.312
**mg/w MTX** *** **dose mg/w**	.038	.256	.026	.148	.883

* Disease Activity Score-Erythrocyte Sedimentation Rate

** Body mass index

*** Methotrexate.

## Discussion

In the present study, we examined skin reactions to capsaicin as an indicator of substance P release in RA patients and compared it with healthy controls. 

The results of this study showed that area of ​​redness and indurated area after applying capsaicin on the forearm, were not significantly different between RA patients and the control group. However, time to tingling was shorter in the control group than the newly diagnosed RA patients.

 Although some studies did not show increased sensitivity to capsaicin in RA (Cervero and Plenderleith, 1987 ▶) , some others have suggested increased capsaicin reactions in RA patients  (Caterina et al., 2000 ▶; Cruwys et al., 1995 ▶; Morris, Cruwysand Kidd, 1997 ▶) .

It has been reported that capsaicin induces vasodilation in the skin overlying joints in RA patients(Jolliffe et al., 1995 ▶) . Most of the previous studies have suggested that maximal reaction occurs when the joint is inflamed, and it seems that the severity of reaction reduces by increasing distance from the inflamed joint (Morris et al., 1997 ▶) . The role of receptors at different sites should be considered when discussing clinical features of RA.

According to our study, in capsaicin test, RA patients experienced tingling at a later time than healthy controls which may indicate the depletion of sensory nerves from substance P in the process of inflammation. The question is whether the peripheral neurogenic inflammation alone can contribute to inflammatory process of chronic diseases such as RA.

In Wayne et al. study, despite high serum levels of substance P in RA patients, no significant association was found between the duration of illness and laboratory data of inflammation; however, the pathogenic role of other mechanisms should be considered (Wayne Marshall et al., 1990 ▶) .

As far as animal and human studies are concerned, most of them have shown that reaction severity does not correlate with the severity of joint inflammation (Cruwys et al., 1995 ▶) . In our findings, primary analysis suggested that redness areas after 15 and 20 min were larger in inactive RA patients. However, regression analysis showed that only disease duration was related to skin reaction according to final redness area. Previously diagnosed RA patients showed more exaggerated skin redness, thus, it might be concluded that disease activity is not correlated with substance P release from the skin neuroendings, but previously diagnosed RA patients may have more substance P supplies in nerve-endings.

The theory of increased number of receptors sensitive to capsaicin and their up regulation in autoimmune diseases, can be used to explain the release of neurotransmitters involved in inflammation in previously diagnosed RA patients (Engler et al., 2007 ▶) .

It has been suggested that after the administration of capsaicin, substance P release from the central and peripheral terminals of primary afferents decrease (Patowary et al., 2017 ▶) . Therefore, the role of the CNS should be considered in chronic forms of RA patients (McInnesand Schett, 2017 ▶) . 

The results of the studies done by Simone et al. and Markenson et al. are in line with the present findings, which did not show a significant difference in time to tingling and mean redness area between the patient and control groups, suggesting the role of signals originating from the spinal cord and the CNS. However, there is a hypothesis that mismatch of the release of substance P in joints and skin is due to the lack of consistency in skin reserves in comparison with joints. Moreover, the greater distance from inflamed joint is associate with less marked skin response to capsaicin (Markenson, 1996 ▶; Simone et al., 1991 ▶) .

The role of drugs in response to capsaicin is challenging. NSAIDS have been introduced as drugs that reduce response to capsaicin (Sacerdote, 1995 ▶; Bakir, 2016); however, Herbert et al. showed that response to capsaicin is not suppressed by cyclooxygenase inhibitors (Herbert, 1993). In our study, primary analysis showed a negative association between drugs and capsaicin skin test. However, regression analysis did not show any association between treatment strategies and capsaicin skin test. It should be mentioned that in our study, none of the patients were receiving NSAIDS. 

The methods used form measuring skin reactions and sample selection according to disease duration, were the strengths of this study. However, this study had some limitations. First, we could not stop treatment before testing. Second, high doses of capsaicin solution were not used because of severe skin reactions observed in the pilot study. 

This study showed no difference in skin hypersensitivity to capsaicin in RA patients when compared to healthy controls. Comparison of previously diagnosed RA patients and newly diagnosed cases revealed a moderate increase in the redness area in the previously diagnosed RA patients, suggesting increased substance P levels in these patients. Given the importance of TRPV1 receptors and substance P in joint inflammation, further studies are required to determine the precise role of capsaicin and substance P in RA development.
